# Preparation and Characterization of Polypropylene/Carbon Nanotubes (PP/CNTs) Nanocomposites as Potential Strain Gauges for Structural Health Monitoring

**DOI:** 10.3390/nano10040814

**Published:** 2020-04-24

**Authors:** Bartolomeo Coppola, Luciano Di Maio, Loredana Incarnato, Jean-Marc Tulliani

**Affiliations:** 1 Department of Applied Science and Technology (DISAT), Politecnico di Torino, INSTM R.U. Lince Laboratory, Corso Duca Degli Abruzzi 24, 10129 Torino, Italy; jeanmarc.tulliani@polito.it; 2 Department of Industrial Engineering (DIIN), University of Salerno, Via Giovanni Paolo II 132, 84084 Fisciano (SA), Italy; ldimaio@unisa.it (L.D.M.); lincarnato@unisa.it (L.I.)

**Keywords:** polypropylene, carbon nanotubes, nanocomposites, strain gauges, structural health monitoring

## Abstract

Polypropylene/carbon nanotubes (PP/CNTs) nanocomposites with different CNTs concentrations (i.e., 1, 2, 3, 5 and 7 wt%) were prepared and tested as strain gauges for structures monitoring. Such sensors were embedded in cementitious mortar prisms and tested in 3-point bending mode recording impedance variation at increasing load. First, thermal (differential scanning calorimetry (DSC), thermo-gravimetric analysis (TGA)), mechanical (tensile tests) and morphological (FE-SEM) properties of nanocomposites blends were assessed. Then, strain-sensing tests were carried out on PP/CNTs strips embedded in cementitious mortars. PP/CNTs nanocomposites blends with CNTs content of 1, 2 and 3 wt% did not show significant results because these concentrations are below the electrical percolation threshold (EPT). On the contrary, PP/CNTs nanocomposites with 5 and 7 wt% of CNTs showed interesting sensing properties. In particular, the best result was highlighted for the PP/CNT nanocomposite with 5 wt% CNTs for which an average gauge factor (GF) of approx. 1400 was measured. Moreover, load-unload cycles reported a good recovery of the initial impedance. Finally, a comparison with some literature results, in terms of GF, was done demonstrating the benefits deriving from the use of PP/CNTs strips as strain-gauges instead of using conductive fillers in the bulk matrix.

## 1. Introduction

Cement-based mortars and concretes are the most used material on Earth (4.1 Gt of cement were produced in 2017, according to Cembureau estimations [1]) and are responsible for about 4% of overall anthropic carbon dioxide emissions (1.46 ± 0.19 GtCO_2_ were released in 2016 due to cement production [2]). For these reasons, researchers are finding more eco-sustainable solutions such as the reuse of construction and demolition wastes (CDW), alkali-activation of waste materials, use of natural fibers instead of synthetic ones, natural aggregates substitution, self-healing materials, etc. [3,4,5,6,7,8,9]. However, while structural properties of the built environment have been deeply studied, relatively less attention has been dedicated to its functional properties and durability monitoring.

Indeed, in the last decades, structural health monitoring (SHM), i.e., systems and structures that can monitor their own structural integrity, attracted popularity in the civil engineering community [10,11,12,13]. Compared to conventional techniques such as in-situ inspections or non-destructive testing (NDT), strain-sensitive sensors represent the latest developed smart systems for structures. This self-monitoring ability is achieved by correlating the change in the applied loads with electrical resistance or impedance variations of the material. Then, the stress or stress state can be estimated allowing the continuous monitoring of damage or microfractures behavior.

Many authors investigated the possibility to load cement-based materials with electrically conducting particles, such as carbon fibers [14,15], steel fibers [16], carbon black [17], nickel powder [18] or carbon nanotubes (CNTs) [19,20,21,22,23,24,25]. In particular, the remarkable mechanical and electrical properties of CNTs suggest that they are ideal candidates for creating smart nanocomposites both in cementitious and polymeric materials.

However, even if the electrical conductivity of cementitious materials is significantly increased while maintaining or even improving their mechanical properties, their implementation is limited due to several environmental factors (such as humidity and temperature) affecting the measurement [26,27,28,29,30]. Moreover, the huge amount of fillers to be used in the bulk matrix as well as, the potential toxicity of functionalized CNTs when mixed on-site [31], discourages their diffusion. For this reason, a viable strategy can be the use of polymer/CNTs nanocomposites as sensing materials. Indeed, several polymer/CNTs nanocomposites have been previously investigated and tested as strain-sensors, exploring the use of different polymer matrices: epoxy resin [32,33,34], polymethyl methacrylate [35] and polypropylene [36,37,38,39,40,41,42]. Polymer/CNTs are excellent sensing materials if the electrical percolation threshold (EPT) is exceeded. Among the previously cited polymers, polypropylene (PP) is one of the most used polymer thanks to its good mechanical properties and chemical resistance (i.e., PP fibers are chemically inert into the alkaline environment typical of cementitious materials). In particular, polypropylene fibers are generally used to avoid shrinkage cracking phenomena or concrete spalling [43,44,45,46]. However, one of the main concerns in PP/CNT nanocomposites preparation is CNTs dispersion in the polymer matrix that, if not properly performed, significantly influences polymer/CNT nanocomposite properties, particularly mechanical and electrical properties. To overcome this drawback, several strategies have been adopted such as processing parameters variation, use of a low-molecular weight polymer and CNTs functionalization [47,48,49,50]. Furthermore, an important advantage in the preparation of PP/CNT strain-sensors lies in their flexibility. As reported elsewhere, it is important to have flexible strain-sensors in order to be easily used in several fields of application [51,52].

Even if several authors investigated sensing ability of PP/CNT alone [36,37,38,39,40,41,42], to the best of our knowledge, no studies investigated the possibility to use PP/CNT strain sensors directly embedded in cementitious materials. Several authors proposed the use of sensing skins or sensing elements to be applied on external surfaces of buildings/infrastructures [53,54,55,56,57]. Nevertheless, the application of sensing skins requires specialized technicians and constant maintenance due to durability issues, increasing the cost and delaying the entry into service of the building or the infrastructure.

Finally, very recently, numerous authors investigated the possibility to prepare conductive elements made with polymer nanocomposites via dry printing [58,59], inkjet printing [60,61] or 3D printing [62,63,64,65]. However, while such systems require new processing instruments, in some cases very expensive, polymer extrusion is easier and can keep low the costs of the produced parts, resulting to be useful for large scale applications such as building and construction sector.

The aim of this work was to produce PP/CNT sensors to be used in the field of SHM of cementitious structures. In particular, the novelty of this work lies on the easy and safe preparation of cheap strain gauge sensors for SHM. Indeed, as already underlined, most of the previous works in the field of SHM for cementitious materials investigated the possibility to use CNTs or other conductive fillers in bulk, posing several risks for the human health that discourages their use. In this work, PP/CNTs blends (1, 2, 3, 5 and 7 wt%, respectively) were prepared via a melt-compounding process by using a twin-screw extruder. Then, PP/CNT nanocomposite strips were extruded with a single-screw extruder and embedded in a cementitious mortar. Finally, prismatic mortar samples containing PP/CNT nanocomposite strips were submitted to measurements of electric impedance under load during three points bending tests.

## 2. Materials and Methods

### 2.1. Materials

Polypropylene Hi-Prene M710 (GS-Caltex, Seoul, South Korea) was used as matrix. A conductive PP-based masterbatch (PLASTICYL PP2001) containing multiwalled CNTs (MWCNT, 20 wt%, Nanocyl NC7000) was used for composites preparation (Nanocyl, Sambreville, Belgium). According to Nanocyl datasheets, the average diameter, the average length and the carbon purity of NC7000 MWCNT are 9.5 nm, 1.5 μm and 90%, respectively.

### 2.2. Preparation of PP/CNT Composites

PP/CNT composites were prepared via melt-compounding using a twin-screw extruder (Dr. Collin GmbH-ZK 25-48D, Ebersberg, Germany) operating at 150 rpm with a flat temperature profile of 210 °C. PP matrix and PP2001 pellets were opportunely mixed to dilute the initial CNT concentration (i.e., 20 wt%) to five fixed concentrations: 1, 2, 3, 5 and 7 wt%, respectively. Therefore, PP/CNT composites nomenclature indicates MWCNT concentration (e.g., PP/CNT-5 is the mixture containing 5 wt% of MWCNT). PP/CNT pellets were produced using a laboratory pelletizer from the extruded strand which was quenched using a cold water bath.

PP/CNT strips were prepared with the PP/CNT pellets using a single-screw extruder (Brabender Do-Corder E330, D_screw_ = 20 mm, L/D = 20, Duisburg, Germany) equipped with a slit die of 1 × 30 mm^2^. The extrusion was carried out with a flat temperature profile of 200 °C and a screw speed of 20 rpm. Strips were collected on a take up system with air cooling to obtain a final thickness and width of 0.8 and 25 mm respectively. Then, as-extruded strips were cut for mechanical and impedance measurements tests.

### 2.3. PP/CNT Composites Characterization

Thermogravimetric analysis (TGA) was carried out on PP and PP/CNT from 25 to 800 °C under nitrogen atmosphere (50 mL/min) at 10 °C/min (Mettler TC-10 Mettler-Toledo, Columbus, OH, USA). Differential scanning calorimetry (DSC) was performed on PP and PP/CNT strips (DSC 822e, Mettler-Toledo, Columbus, OH, USA) performing the following thermal cycle under nitrogen atmosphere: a first heating at 10 °C/min from −50 °C to 200 °C, a dwell at 200 °C for 10 min, a cooling down to −50 °C and a second heating up to 200 °C at the same heating rate. The degree of crystallinity, Xc, of the different samples was calculated by the following equation (Equation (1)):

(1) $$Xc\left(\%\right)=\frac{\Delta H_{m}}{\left(1-\phi \right)\Delta H_{100}}\cdot 100$$

where Δ*H_m_* is the heat of crystallization of the analyzed sample (J/g), Δ*H*_100_ is a reference value that represents the heat of crystallization for a 100% crystalline polymer (209 J/g for a 100% crystalline PP [36,66]), and *ϕ* is the CNT weight percentage.

Tensile tests were conducted on rectangular strips (12 cm long having a gauge length of 5 cm) according to ASTM D638 and ISO 527-1 using a universal testing machine (Sans CMT6000 series, Shenzhen City, China) equipped with a load cell of 1 kN. Tensile tests were performed at two crosshead speeds (5 and 100 mm/min) for determining the elastic modulus (E) and properties at failure (stress at break, σ_b_, and strain at break, ε_b_). Mechanical properties are the average of at least ten measurements conducted for each composition.

X-ray Diffraction (XRD) analysis was performed on strips surface and spectra were recorded using a Pan’Analytical X’Pert Pro instrument (Pan’Analytical, Almelo, The Netherlands) with CuKα radiation (λ = 0.154056 nm) in the 2θ range 10–30°. PP/CNT morphology was investigated using a FE-SEM (Zeiss Supra-40, Oberkochen, Germany) after sputtering samples with a thin coating of chromium.

### 2.4. Mortar Samples Preparation

**Figure 1:**
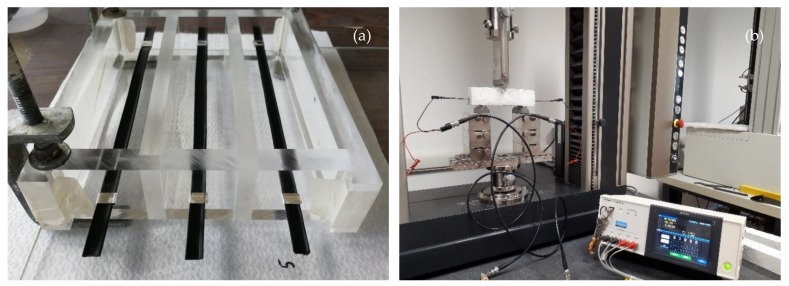
(**a**) Polypropylene/carbon nanotubes (PP/CNT) strips in the PMMA mold and (**b**) impedance measurement setup.

PP/CNT strips were embedded in a cementitious mortar prepared according to EN 196-1 using a standard silica sand, Portland cement (CEM II/B-LL 32.5 R) and deionized water with an aggregate, cement, water proportion of 3:1:0.5. In particular, PP/CNT strips were located in the inferior part (1/3 from the bottom, where tensile stresses are higher) of a PMMA mold (Figure 1a) before mortar casting. Prismatic specimens (40 mm × 40 mm × 160 mm) were then wrapped in a closed bag for one day, demolded and cured in water for 28 days at room temperature and air dried in the lab for 1 week before resistivity tests. Three samples for each PP/CNT mixture were tested.

### 2.5. Strain-Sensing Tests

Self-sensing ability of PP/CNT composites were determined using a 3-point bending test on mortar samples containing PP/CNT strips (Figure 1b). In particular, bending tests were carried out using a universal testing machine (Zwick Roell 2014, Ulm, Germany) equipped with a load cell of 50 kN at a strain rate of 0.25 mm/min. Tests were stopped at a maximum load of 1500 N to avoid sample failure and allow repetition of tests. Indeed, each sample was loaded and unloaded three times to verify measurements reproducibility and possible decay of the self-sensing ability over the time. Specimens strain (ε) was calculated according to [67], using Equation (2):

(2) $$\varepsilon =-\frac{My}{EI}$$

where *M* is the flexural moment (kNm), *y* is the distance from the specimen centroid to the tension surface (m), *E* is the Young’s modulus (GPa), and *I* is the moment of inertia of the specimen cross section (m^4^). PP/CNT resistivity was assessed considering impedance variation (Δ*Z*) under load. Impedance, *Z*, was measured using an impedance analyzer (LCR meter IM3533-01, HIOKI EE. Corp., Nagano, Japan) imposing a ΔV of 1 V and a frequency of 5 kHz (Figure 1b). At this working frequency, the instrument used guaranties an accurate measurement of the impedance in the range 10 mΩ–200 MΩ. The fractional change in electrical impedance, Δ*Z_f_*, was calculated as the ratio between impedance variation, due to the applied strains, and initial impedance, according to Equation (3):

(3) $$\Delta Z_{f}(\%)=\frac{\Delta Z}{Z_{0}}\cdot 100$$

Finally, the Gauge Factor (GF) was calculated as following (Equation (4)):

(4) $$\mathrm{GF}=\frac{\Delta Z_{f}}{\varepsilon }$$

In this work, impedance was measured instead of resistance, to eventually detect capacitive effects [68]. However, the phase angle was always below 5°, indicating that the CNTs rather behave as pure resistors than capacitors.

## 3. Results

### 3.1. PP/CNT Composites Characterization

#### 3.1.1. XRD Measurements

**Figure 2:**
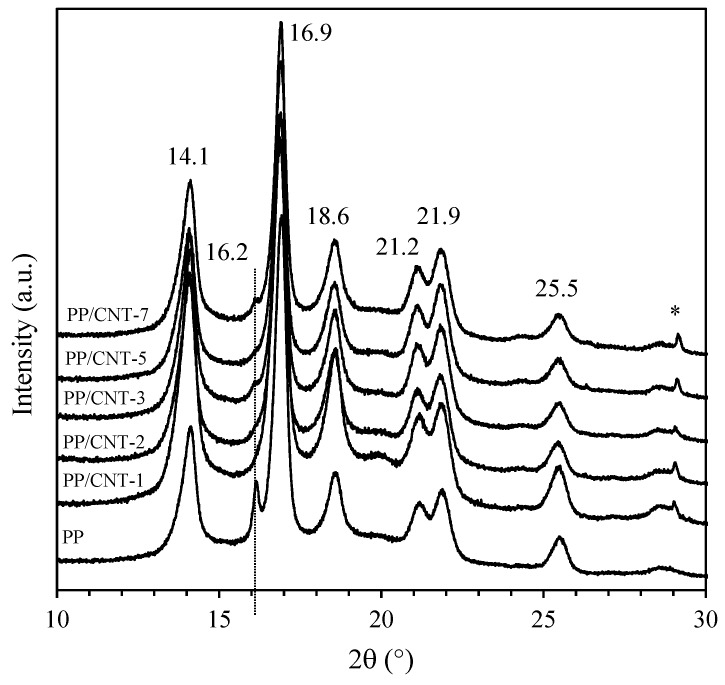
XRD patterns of PP and PP/CNT nanocomposites in the 10–30° 2θ range (* is a peak corresponding to catalyst residue).

**Figure 3:**
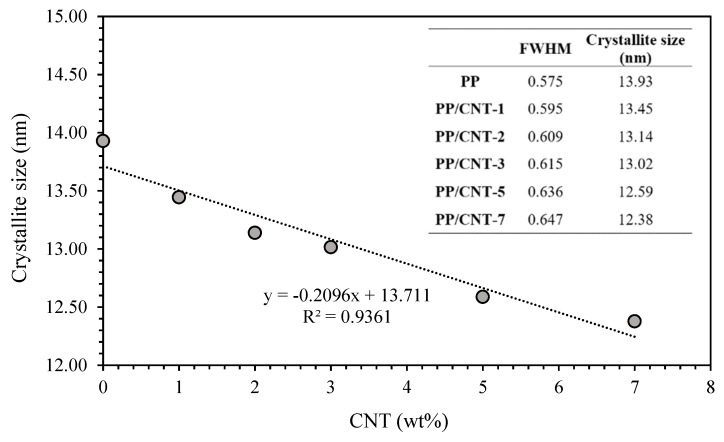
Crystallite size determined using Scherrer’s equation for the lattice plane (110) at 14.1°.

XRD patterns of neat PP and PP/CNT composites are reported in Figure 2. Both neat PP and PP/CNT composites show the reflections characteristic of the crystalline α-form of PP at 2θ of 14.1° (110), 16.9° (040), 18.6° (130), 21.2° (111), 21.9° (131) and 25.5° (060) [36,69,70]. In addition, neat PP shows an additional peak at 2θ of 16.2° representative of the β-form (300) that disappears for PP/CNT composites. Indeed, it is well known that CNTs promote crystallization of the more stable α-form [71], as also evident from the increase of the intensity of the peaks corresponding to this crystalline phase. Moreover, to better analyze the influence of CNTs on the crystallographic morphology, full width at half-maximum (FWHM) values were determined for the peak at 14.1° 2θ corresponding to the lattice plane (110). At increasing CNTs content FWHM increases and, according to Scherrer’s equation, the average thickness of crystallites decreases (Figure 3). Das and Satapathy [72] attributed this trend to the nucleation effect exerted by CNTs because a faster nucleation process facilitates crystallite size reduction and monoclinic α-form formation.

#### 3.1.2. Thermal Properties

The influence of CNTs on PP thermal properties (melting temperature, T_m_, enthalpy of fusion, Δ*H_m_*, and degree of crystallinity, Xc) were investigated using DSC and results are reported in Table 1 and Table 2 for the I and II heating, respectively. Melting temperature slightly decreases for PP/CNT nanocomposites compared to neat PP (approximatively 2 °C) due to a decrease of crystallites size, as previously discussed (Section 3.1.1). Coherently, T_m_ of Plasticyl is even lower due to the higher content of CNTs (i.e., 20 wt%).

With regard to the melting enthalpies (Δ*H_m_*), no dependency on the CNTs content can be recognized (Table 1). However, using these values to calculate the degree of crystallinity (Xc) a slight increase of Xc was measured for PP/CNT nanocomposites, compared to neat PP (Table 1). Considering thermal properties of the II heating (i.e., after removal of the thermal history due to processing conditions), no meaningful variations can be distinguished among the investigated samples in terms of melting temperature that is approx. 168 ± 1 °C (Table 2). On the contrary, crystallinity degree of PP/CNT nanocomposites is significantly higher compared to the one of neat PP. Firstly, the influence of processing conditions is removed during the II heating, meaning that the influence of ribbon drawing and air cooling during extrusion on the degree of crystallinity are removed. Then, the nucleation effect of CNTs leads to a constant degree of crystallinity for PP/CNTs nanocomposites, independently to the CNTs loading (Table 2). Indeed, even for the masterbatch (i.e., at the highest CNTs loading, 20 wt%) Xc is approx. the same as for the other PP/CNT nanocomposites. Similarly, Wang et al. [36], reported a progressive increase of the degree of crystallinity of PP/CNT nanocomposites up to CNTs concentrations of 1.5 wt% with a slight increase between 1.5 and 2.5 wt%.

**Figure 4:**
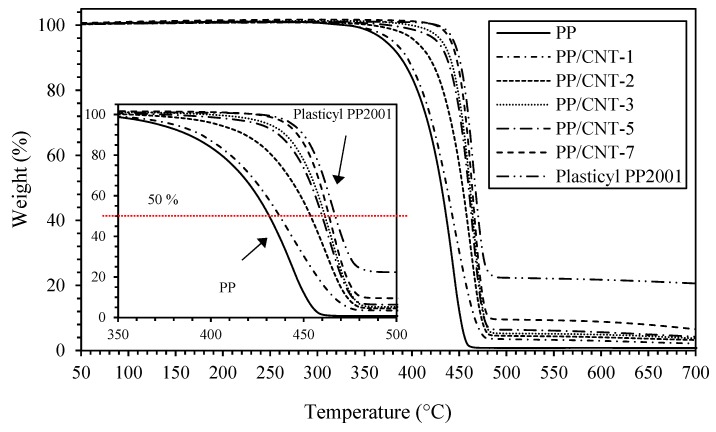
Weight loss of PP and PP/CNT nanocomposites (detail in the range 350–500 °C in the inset).

TGA analyses were carried out to evaluate the effect of CNTs addition to PP thermal stability and degradation. Figure 4 reports degradation curves while in Table 3 the degradation onset temperature (T_ONSET_) and temperatures corresponding to a weight loss of 25, 50 and 75% respect to the initial weight (T_25_, T_50_ and T_75_, respectively) are indicated. As evident, at increasing CNTs content the T_50_ is shifted towards higher temperatures, compared to the neat PP (inset of Figure 4). In particular, T_50_ for neat PP is 431 °C and increases up to 464 °C for PP/CNT-7 corresponding to an increase of T_50_ of approx. 30 °C (Table 3). Finally, the highest increase was measured for the masterbatch (i.e., Plasticyl PP2001) that contains 20 wt% of CNTs. The sharp increase of thermal stability can be related to the barrier effect exerted by CNTs against degradation products transport, similarly to what already observed for other polymer nanocomposites [73,74].

#### 3.1.3. Mechanical Properties

**Figure 5:**
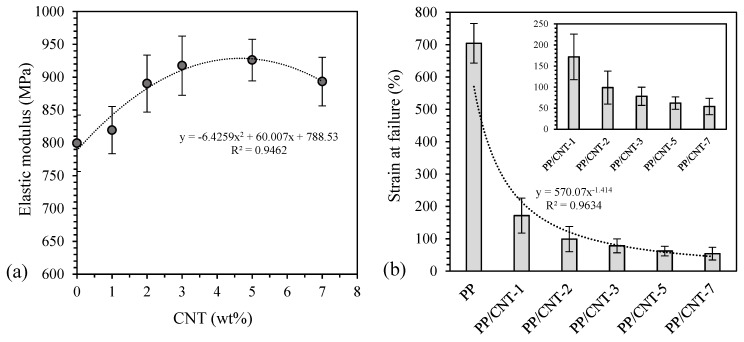
(**a**) Elastic modulus and (**b**) strain at failure as a function of carbon nanotubes (CNTs) wt% content.

Mechanical properties, in terms of elastic modulus (E), stress and strain at yield (σ_y_ and ε_y_, respectively), stress and strain at failure (σ_b_ and ε_b_, respectively) of neat PP and PP/CNT composites are reported in Table 4. Stress-strain curves have a similar trend but CNTs significantly influence mechanical properties, in particular elastic modulus and strain at failure (E and ε_b_, respectively). Indeed, at increasing CNTs content, an increase of elastic modulus was measured up to 5 wt% of CNTs content (Figure 5a) coherently to what elsewhere reported for polymer nanocomposites [66,75]. Then, a further increase of the CNTs concentration leads to a decrease of this property, as evident from the fitting proposed in Figure 5a. Elastic modulus slightly increases at 1 wt% of CNTs content (+2%) while a more important increase was measured for higher CNTs loadings (+11%, 15% and 16% for 2, 3 and 5 wt% addition, respectively). Stress and strain at yield were not significantly affected by CNTs addition, reporting only a slight increase of stress at yield (Table 4). Also tensile strength was approximatively the same for PP and PP/CNTs nanocomposites (Table 4) while elongation at rupture sharply decreases at increasing CNTs content (Figure 5b), as well known in the literature [66].

#### 3.1.4. FE-SEM Micrographs

**Figure 6:**
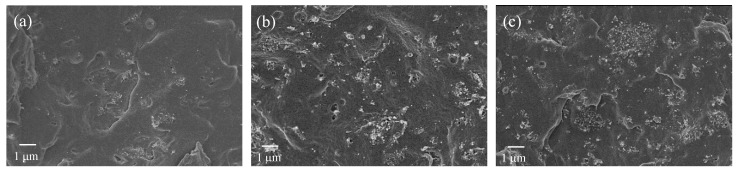
(**a**) PP/CNT-3, (**b**) PP/CNT-5 and (**c**) PP/CNT-7.

**Figure 7:**
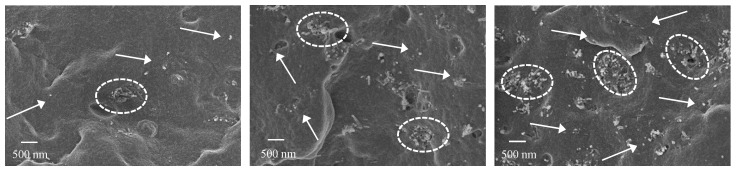
(**a**) PP/CNT-3, (**b**) PP/CNT-5 and (**c**) PP/CNT-7. White arrows indicate single dispersed CNTs; white ellipsis indicate CNTs agglomerates.

CNTs distribution and dispersion in PP/CNT nanocomposites were investigated using a FE-SEM. Micrographs took on cryogenically fractured surface of PP/CNT-3, PP/CNT-5 and PP/CNT-7 are reported in Figure 6 and Figure 7. At lower magnifications (Figure 6) it is possible to observe a good distribution of CNTs in the PP matrix and the progressive increase of CNTs amount (i.e., 3, 5 and 7 wt%) can be easily appreciated moving from Figure 6a to Figure 6c. However, at higher magnifications (Figure 7), several agglomerates (indicated with white ellipsis in Figure 7) can be recognized, in particular for PP/CNT-7 nanocomposites (Figure 7c). Nevertheless, some dispersed tubes are also visible between these agglomerates (white arrows in Figure 7). As reported elsewhere [36,48,49], CNTs agglomerates in the order of 500 nm are generally found and one strategy to reduce CNTs tendency to agglomerates is the use of low molecular weight polymers. As evident in Figure 6, only in the case of the nanocomposite PP/CNT-7 agglomerates bigger than 1 μm can be found and such morphology can explain the decrease of mechanical properties measured for this nanocomposite (Figure 5a). Moreover, the presence of agglomerates can also explain the progressive decrease of elongation at break measured for PP/CNT nanocomposites compared to neat PP (Figure 5b).

**Figure 8:**
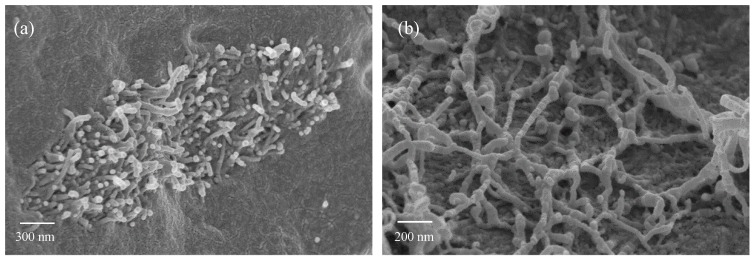
(**a**) CNTs agglomerate and (**b**) detail of CNTs.

In Figure 8 two detail micrographs showing a CNTs agglomerate (Figure 8a) and CNTs spatial dispersion in the polymer matrix (Figure 8b) are reported. No interfacial transition zone (ITZ) can be recognized between CNTs and PP (Figure 8a) meaning a good wetting of CNTs by PP, responsible of the good mechanical properties obtained for PP/CNT nanocomposites. At higher magnifications (Figure 8b), it is evident that CNTs are homogenously dispersed in the PP matrix even if they are entangled and curved. No CNTs were found on PP/CNT strips surfaces (micrographs not reported) due to the manufacturing process (i.e., the extrudate in a semi-molten phase is partially stretched and pressed by the take up system to confer the required shape). However, this is positive both in terms of safe handling and in terms of influence of environmental conditions on sensors response. With regard to safety, this means that no CNTs are exposed and all of them are embedded in the polymer matrix, excluding any possible dispersion in the air. Moreover, even if the cementitious substrate is wet or contains ions (for example deriving from de-icing salts), sensors electrical response is not influenced by environmental conditions. On the contrary, when CNTs are directly immersed in the bulk cementitious matrix, the surrounding environment directly influences sensing ability [26,27,28,29].

### 3.2. Strain-Sensing Tests

**Figure 9:**
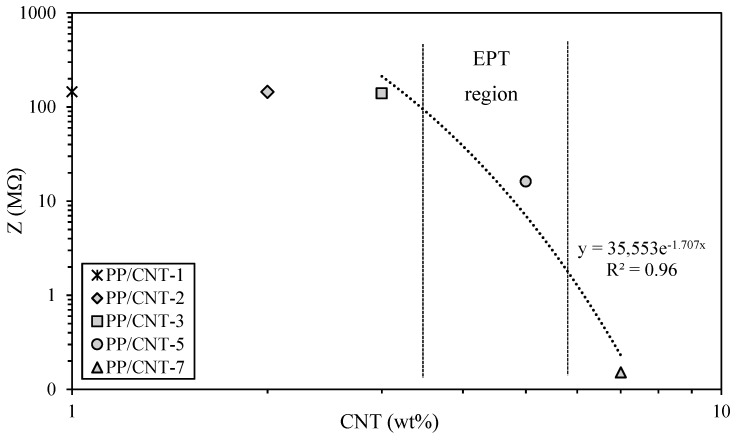
Impedance of the different PP/CNT nanocomposites strips.

Impedance of the different PP/CNT nanocomposites was first measured using a two-probe LCR meter and the results are reported in Figure 9. As evident, no meaningful impedance variations are recorded up to PP/CNT-5 nanocomposite, thus EPT should be comprised between 3 and 5 wt% of CNTs. Even if lower EPT values (approx. 1 wt% of CNTs) are reported elsewhere for similar formulations [76,77], processing parameters, fabrication route and CNTs alignment greatly influence electrical properties of polymer/CNTs nanocomposites [78,79]. Indeed, comparable results were found by Müller et al. [48] for polyethylene (PE) nanocomposites prepared with similar processing parameters (i.e., twin screw micro-compounder, 200 °C and 200 rpm). Authors reported that the EPT is above 2.5 wt% of CNTs loading and that the plateau region is not reached up to 6.0 wt% [48]. Accordingly, in our study a sharp impedance decrease was measured above 3 wt% (Figure 9). At these CNTs weight fractions an elevated number of CNTs is homogeneously distributed in the PP matrix, as discussed before (Figure 6). Therefore, impedance was measured only on mortar samples containing PP/CNT-3, PP/CNT-5 and PP/CNT-7 strips.

**Figure 10:**
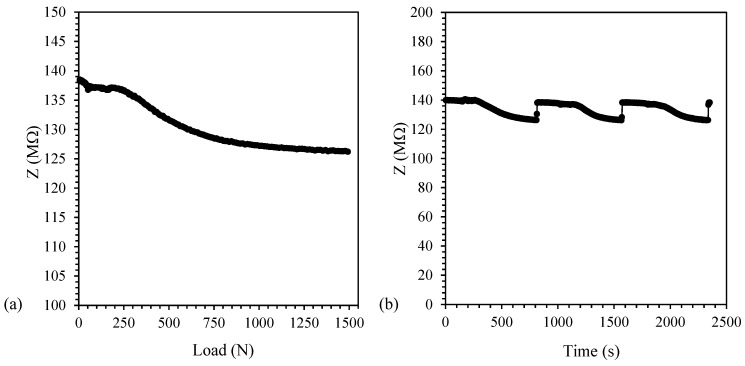
Impedance value for PP/CNT-3 nanocomposites. (**a**) Impedance (Z) variation vs. load and (**b**) load/unload cycles.

**Figure 11:**
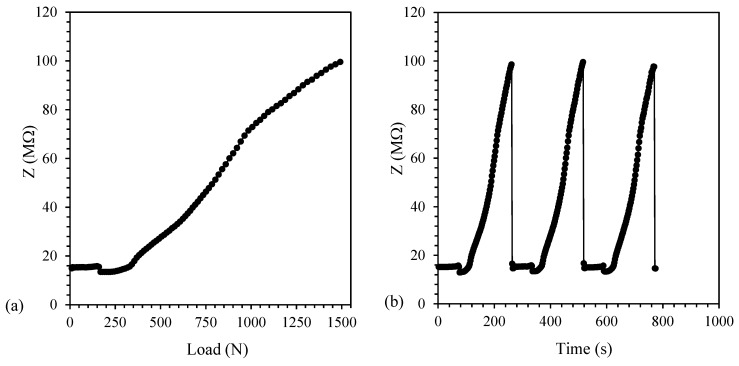
Impedance value for PP/CNT-5 nanocomposites. (**a**) Impedance (Z) variation vs. load and (**b**) load/unload cycles.

**Figure 12:**
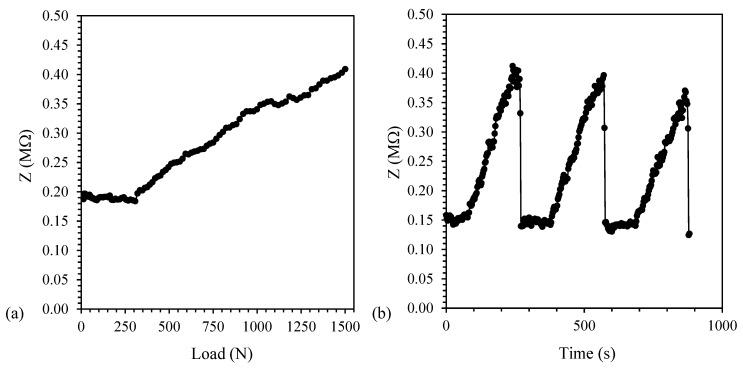
Impedance value for PP/CNT-7 nanocomposites. (**a**) Impedance (Z) variation vs. load and (**b**) load/unload cycles.

The results of the impedance measurements on PP/CNT-3, PP/CNT-5 and PP/CNT-7 samples are shown in Figure 10, Figure 11 and Figure 12, respectively. PP/CNT-3 nanocomposites reported an impedance decrease under load, starting from approx. 250 N and then exhibited a constant value after approx. 1000 N (Figure 10a). This behavior can be explained considering that EPT is not yet reached (as observed in Figure 7a) and once the load is applied, CNTs reorganization occurs due to the applied strain and Poisson’s ratio [32]. This modification in the CNTs spatial morphology leads to a slight decrease of impedance for PP/CNT-3 nanocomposites (Δ*Z* = 13 MΩ, considered as absolute value, Table 5) because CNTs tend to get closer under load. Repeated load/unload tests were done to verify measurement repeatability and the results are reported in Figure 10b. As evident, even after three tests, the starting impedance is recovered when the sample is unloaded meaning a good reproducibility of the test and that PP/CNT nanocomposites are not damaged after testing. 

On the contrary, above EPT (i.e., for PP/CNT-5 and PP/CNT-7 nanocomposites, Figure 11 and Figure 12, respectively) a different behavior is recognizable. In these nanocomposites, the applied strain and Poisson’s ratio disentangle CNTs agglomerates and increase the distance between single CNTs. In the case of PP/CNT-5 nanocomposites, i.e., in the EPT region (Figure 9), the variation of the conductive network results in a very high impedance variation (Δ*Z* = 113 MΩ, Table 5). This result can be explained considering the contextual modification of the distance between adjacent CNTs, affecting the tunneling effect, and the loss of contacts in CNTs agglomerates. Indeed, in the case of small strains, the tunneling effect determines electrical properties variations [32]. Finally, because of the very high CNTs content in the PP/CNT-7 nanocomposite, even if the conductive network experience some modifications, the impedance variation is very low (Δ*Z* = 0.26 MΩ, Table 5). 

With regard to repeated tests, also in the case of PP/CNT-5 and PP/CNT-7 nanocomposites, the reproducibility of the measurement and the recovery of the initial impedance value after load removal were confirmed (Figure 11b and Figure 12b, respectively), contrarily to what reported by Naeem et al. [67] for MWNT/cement composites. Indeed, even small strains are able to rearrange CNTs fillers distribution in cementitious materials, on the contrary, the advantage in the use of PP/CNT nanocomposites strips, is represented by the use of a flexible material that recover its original properties. 

Table 5 summarizes strain-sensing tests results in terms of impedance variation (Δ*Z*) and fractional change in electrical impedance (Δ*Z_f_*). As stated before, PP/CNT-5 nanocomposites reported the highest Δ*Z* (113 ± 34 MΩ) and, consequently, Δ*Z_f_* (692 ± 73%), at the maximum reached load (i.e., 1500 N), compared to the other investigated nanocomposites (i.e., PP/CNT-3 and PP/CNT-7, Table 5). According to the literature [32,35], higher sensitivity is obtained for nanocomposites near EPT, confirming what previously stated.

**Figure 13:**
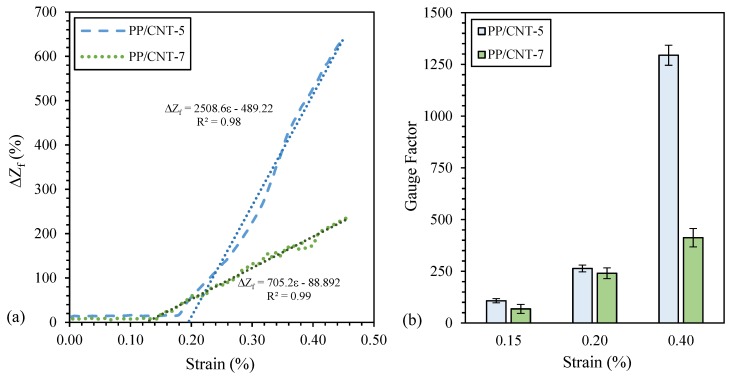
(**a**) fractional change in electrical impedance (Δ*Z_f_*) as a function of strain and (**b**) Gauge Factor (GF) at fixed strains (i.e., 0.15%, 0.20% and 0.40%, respectively) for PP/CNT-5 and PP/CNT-7 nanocomposites.

The average GF at the maximum achieved strain (approx. 0.55%) of the investigated nanocomposites was calculated according to Equation (4) and the determined values are reported in Table 5. Moreover, the relationship between the fractional change in electrical impedance (Δ*Z_f_*) and the strain for two representative samples is reported in Figure 13a. As evident, a strong linear relationship exists between Δ*Z_f_* and the strain above the minimum value of strain necessary to detect the strain variation. In particular, such value is slightly lower for PP/CNT-7 nanocomposites (approx. 0.13%) compared to PP/CNT-5 strips (approx. 0.19%). Then, above this strain detection threshold, PP/CNT-5 has a considerably higher GF compared to PP/CNT-7 nanocomposites. In Figure 13b, the GF of PP/CNT-5 and PP/CNT-7 strips at fixed strains (i.e., 0.15%, 0.20% and 0.40%, respectively) are reported. As evident, PP/CNT-5 and PP/CNT-7 have similar GF up to 0.20% of strain (approx. 250 at 0.20%) while a sharp difference is recognizable at 0.40% of strain (approx. 1300 and 400 for PP/CNT-5 and PP/CNT-7, respectively). GF decreases at increasing CNTs contents (Figure 13b), as reported also elsewhere [32], but linearity (i.e., *R*^2^) slightly increases (Figure 13a).

**Figure 14:**
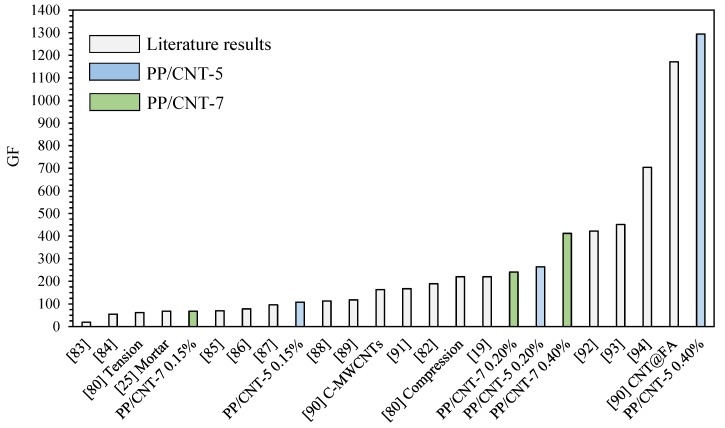
Comparison of the GF of different strain-sensing cementitious materials.

A comparison of the results, in terms of GF, obtained in this work with some papers dealing with strain-sensing cementitious materials are reported in Figure 14. However, a direct comparison is difficult because the strain sensing ability is affected by several parameters: testing mode (compression and tension) [80], load rate [81], conductive filler (CNF, CNT etc) [82], cementitious material (paste, mortar or concrete) [25], etc. Nevertheless, it is evident that the GF for PP/CNT-5 and PP/CNT-7 at 0.20% of strain is higher than the GF of most of the previous published works here used as comparison (Figure 14) [19,25,80,82,83,84,85,86,87,88,89,90,91]. Moreover, PP/CNT-5 nanocomposites at 0.40% of strain have the highest GF among the compared works (Figure 14) [19,25,80,82,83,84,85,86,87,88,89,90,91,92,93,94]. Finally, it should be considered that the amount of CNTs used in this work is limited compared to the previous published papers investigating the use of conductive fillers in the bulk cementitious matrix.

## 4. Conclusions

In this study, for the first time, strips of PP/CNT nanocomposites prepared via melt-compounding are embedded in a cementitious mortar as potential strain gauges for structural health monitoring. First, a series of PP/CNT nanocomposites (i.e., 1, 2, 3, 5 and 7 wt%) were prepared via melt-compounding in a twin-screw extruder. Then, ribbon-shaped sensors were extruded using a single-screw extruder. The effects of CNTs loading on the thermal (DSC and TGA), mechanical and morphological properties of PP/CNT nanocomposites were investigated. From XRD measurements, it has been found that CNTs promoted crystallization of the more stable α-form as β-form was found only in the neat PP. Moreover, at increasing CNTs content, the average crystallites size decreases, as calculated using Scherrer’s equation. Coherently, melting temperature of PP/CNT nanocomposites slightly decreases compared to neat PP, as measured with DSC analysis. Moreover, a significant increase of crystallinity degree was measured for PP/CNT nanocomposites, respect to the neat PP, during the second heating ramp. TGA reported a very high increase of thermal stability for PP/CNT nanocomposites (e.g., T_50_ increases of approx. 30 °C from the neat PP to PP/CNT-7). CNTs significantly influenced PP/CNTs mechanical properties, in particular elastic modulus and strain at failure. Elastic modulus slightly increased for low CNTs content (+2% for PP/CNT-1) while a higher increase was measured for the other nanocomposites (+11%, 15% and 16% for PP/CNT-2, PP/CNT-3 and PP/CNT-5, respectively). A further increase of CNTs content led to a decrease of elastic modulus but it was still higher than neat PP (+12%). On the contrary, elongation at rupture sharply decreases at increasing CNTs content. FE-SEM micrographs reported a good distribution of CNTs in the PP matrix even if some agglomerates, representative of a limited CNTs dispersion, were evident at increasing CNTs loading. Finally, for the first time, strain-sensing ability of PP/CNT nanocomposites embedded into a cementitious mortar was investigated measuring impedance variation. PP/CNT-1 and PP/CNT-2 did not show significant results because well below the EPT (that is between 3 and 5 wt% of CNTs) while PP/CNT composites with 3, 5 and 7 wt% of CNTs showed very good results. Indeed, impedance started to increase even at very low loads (between 350–450 N) and returned to the initial value once the load was removed. PP/CNT-5 nanocomposites showed the best result with an impedance variation of 113 MΩ corresponding to a Δ*Z_f_* of approx. 690%. Moreover, an evident linear relationship (*R*^2^ of 0.98–0.99) exists between Δ*Z_f_* and the strain (above the strain detection threshold that is approx. 0.13% and 0.19% for PP/CNT-7 and PP/CNT-5, respectively). However, PP/CNT-5 and PP/CNT-7 have similar GF up to 0.20% of strain (approx. 250 at 0.20%) while a sharp difference is recognizable at 0.40% of strain (approx. 1300 and 400 for PP/CNT-5 and PP/CNT-7, respectively). These GF values are well above most of the previous published work investigating the use of conductive fillers in the bulk cementitious matrix.

In conclusion, PP/CNT nanocomposites are cheap potential strain gauge sensors for SHM. Future work will be devoted to the ageing of the proposed sensors when submitted to freeze-thaw cycles as well as to the investigation of their response in function of the temperature and of the samples’ humidity content.

## Figures and Tables

**Table 1:** Melting temperature (T_m_), enthalpy of fusion (Δ*H_m_*) and degree of crystallinity (Xc) of PP, Plasticyl PP2001 and PP/CNT composites as determined during the first heating.

Sample	T_m_ (°C)	Δ*H_m_* (J/g)	X_c_ (%)
PP	169.77	71.39	34
Plasticyl PP2001	165.28	65.53	39
PP/CNT-1	167.84	75.70	37
PP/CNT-2	167.44	75.65	37
PP/CNT-3	167.73	66.12	33
PP/CNT-5	167.66	82.07	41
PP/CNT-7	167.75	72.70	37

**Table 2:** Melting temperature (T_m_), enthalpy of fusion (Δ*H_m_*) and degree of crystallinity (Xc) of PP, Plasticyl PP2001 and PP/CNT composites as determined during the second heating.

Sample	T_m_ (°C)	Δ*H_m_* (J/g)	X_c_ (%)
PP	168.85	48.01	23
Plasticyl PP2001	166.28	67.80	41
PP/CNT-1	168.53	82.21	40
PP/CNT-2	167.47	70.88	35
PP/CNT-3	167.42	71.17	35
PP/CNT-5	168.32	74.55	38
PP/CNT-7	167.70	75.50	39

**Table 3:** Degradation onset temperature (T_ONSET_) and temperatures corresponding to a weight loss of 25, 50 and 75% respect to the initial weight.

Sample	T_ONSET_ (°C)	T_25_ (°C)	T_50_ (°C)	T_75_ (°C)
PP	398	411	431	444
Plasticyl PP2001	440	458	467	478
PP/CNT-1	390	416	437	453
PP/CNT-2	423	437	454	465
PP/CNT-3	437	451	461	469
PP/CNT-5	434	449	460	468
PP/CNT-7	435	455	464	471

**Table 4:** Mechanical properties of PP and PP/CNT nanocomposites as determined via tensile tests (results are the average of ten measurements).

	E (MPa)	σ_y_ (MPa)	ε_y_ (%)	σ_b_ (MPa)	ε_b_ (%)
**PP**	799 ± 43	7.31 ± 0.28	1.10 ± 0.01	20.30 ± 1.00	704 ± 61
**PP/CNT-1**	819 ± 36	7.89 ± 0.37	1.11 ± 0.01	21.01 ± 1.28	172 ± 54
**PP/CNT-2**	890 ± 43	8.44 ± 0.45	1.10 ± 0.01	21.94 ± 0.99	99 ± 39
**PP/CNT-3**	918 ± 45	8.10 ± 0.31	1.03 ± 0.01	19.79 ± 1.10	78 ± 22
**PP/CNT-5**	926 ± 32	8.12 ± 0.22	1.04 ± 0.01	22.46 ± 1.97	62 ± 15
**PP/CNT-7**	893 ± 37	8.13 ± 0.44	1.04 ± 0.01	21.50 ± 2.22	54 ± 20

**Table 5:** Summary of results: min and max impedance value (*Z*_MIN_ and *Z*_MAX_, respectively), impedance variation (Δ*Z*), fractional change in electrical impedance (Δ*Z_f_*) and Gauge Factor (GF).

CNT (wt%)	*Z*_MIN_ (MΩ)	*Z*_MAX_ (MΩ)	Δ*Z* (MΩ)	Δ*Z_f_* (%)	GF
3	126 ±0.03	140 ± 2	13 ± 2	11 ± 1	16.6 ± 0.3
5	16 ± 3.77	129 ± 38	113 ± 34	692 ± 73	1416 ± 28
7	0.15 ± 0.02	0.41 ± 0.03	0.26 ± 0.03	172 ± 33	370 ± 67

## Abbreviations

CNTs	Carbon Nanotubes
DSC	Differential Scanning Calorimetry
EPT	Electrical Percolation Threshold
FE-SEM	Field Emission Scanning Electron Microscopy
GF	Δ*Z_f_*/ε
	Gauge Factor
NDT	Non Destructive Testing
PP	Polypropylene
SHM	Structural Health Monitoring
TGA	Thermo-Gravimetric Analysis
XRD	X-ray Diffraction
ΔZ_f_	Δ*Z*/Z_0_
	Fractional change in electrical impedance
ε	Specimen strain

## References

[1] https://cembureau.eu/media/1716/activity-report-2017.pdf.

[2] Andrew R.M. (2018). Global CO_2_ emissions from cement production. Earth Syst. Sci. Data.

[3] Coppola B., Palmero P., Montanaro L., Tulliani J.-M. (2019). Alkali-activation of marble sludge: Influence of curing conditions and waste glass addition. J. Eur. Ceram. Soc..

[4] Bassani M., Tefa L., Coppola B., Palmero P. (2019). Alkali-activation of aggregate fines from construction and demolition waste: Valorisation in view of road pavement subbase applications. J. Clean. Prod..

[5] Ferrara G., Coppola B., Di Maio L., Incarnato L., Martinelli E. (2019). Tensile strength of flax fabrics to be used as reinforcement in cement-based composites: Experimental tests under different environmental exposures. Compos. Part B Eng..

[6] Corinaldesi V., Moriconi G. (2009). Behaviour of cementitious mortars containing different kinds of recycled aggregate. Constr. Build. Mater..

[7] Restuccia L., Spoto C., Ferro G., Tulliani J.-M. (2016). Recycled Mortars with C&D Waste. Procedia Struct. Integr..

[8] Formia A., Irico S., Bertola F., Canonico F., Antonaci P., Pugno N.M., Tulliani J.-M. (2016). Experimental analysis of self-healing cement-based materials incorporating extruded cementitious hollow tubes. J. Intell. Mater. Syst. Struct..

[9] De Belie N., Gruyaert E., Al-Tabbaa A., Antonaci P., Baeră C., Bajare D., Darquennes A., Davies R., Ferrara L., Jefferson A. (2018). A Review of Self-Healing Concrete for Damage Management of Structures. Adv. Mater. Interfaces.

[10] Lynch J.P., Farrar C.R., Michaels J.E. (2016). Structural health monitoring: technological advances to practical implementations. Proc. IEEE.

[11] Han B., Ding S., Yu X. (2015). Intrinsic self-sensing concrete and structures: A review. Measurement.

[12] Tian Z., Li Y., Zheng J., Wang S. (2019). A state-of-the-art on self-sensing concrete: Materials, fabrication and properties. Compos. Part B Eng..

[13] Wang M.L., Lynch J.P., Sohn H. (2014). Sensor Technologies for Civil Infrastructures, Volume 2: Applications in Structural Health Monitoring.

[14] Chen P.-W., Chung D.D.L. (1993). Carbon fiber reinforced concrete for smart structures capable of non-destructive flaw detection. Smart Mater. Struct..

[15] Lavagna L., Musso S., Ferro G., Pavese M. (2018). Cement-based composites containing functionalized carbon fibers. Cem. Concr. Compos..

[16] Chung D.D.L. (2001). Functional properties of cement-matrix composites. J. Mater. Sci..

[17] Han B., Ou J. (2007). Embedded piezoresistive cement-based stress/strain sensor. Sens. Actuators A Phys..

[18] Han B., Yu Y., Han B., Ou J. (2008). Development of a wireless stress/strain measurement system integrated with pressure-sensitive nickel powder-filled cement-based sensors. Sens. Actuators A Phys..

[19] Materazzi A., Ubertini F., D’Alessandro A. (2013). Carbon nanotube cement-based transducers for dynamic sensing of strain. Cem. Concr. Compos..

[20] Li G.Y., Wang P.M., Zhao X. (2007). Pressure-sensitive properties and microstructure of carbon nanotube reinforced cement composites. Cem. Concr. Compos..

[21] Musso S., Tulliani J.-M., Ferro G., Tagliaferro A. (2009). Influence of carbon nanotubes structure on the mechanical behavior of cement composites. Compos. Sci. Technol..

[22] Reales O.A.M., Filho R.D.T. (2017). A review on the chemical, mechanical and microstructural characterization of carbon nanotubes-cement based composites. Constr. Build. Mater..

[23] Konsta-Gdoutos M.S., Danoglidis P., Falara M.G., Nitodas S.F. (2017). Fresh and mechanical properties, and strain sensing of nanomodified cement mortars: The effects of MWCNT aspect ratio, density and functionalization. Cem. Concr. Compos..

[24] Adresi M., Hassani A., Tulliani J.-M., Lacidogna G., Antonaci P. (2017). A study of the main factors affecting the performance of self-sensing concrete. Adv. Cem. Res..

[25] D’Alessandro A., Rallini M., Ubertini F., Materazzi A., Kenny J.M. (2016). Investigations on scalable fabrication procedures for self-sensing carbon nanotube cement-matrix composites for SHM applications. Cem. Concr. Compos..

[26] Wen S., Chung D.D.L. (2008). Effect of moisture on piezoresistivity of carbon fiber-reinforced cement paste. ACI Mater. J..

[27] Dong W., Li W., Lu N., Qu F., Vessalas K., Sheng D. (2019). Piezoresistive behaviours of cement-based sensor with carbon black subjected to various temperature and water content. Compos. Part B Eng..

[28] Demircilioğlu E., Teomete E., Schlangen E., Baeza F.J. (2019). Temperature and moisture effects on electrical resistance and strain sensitivity of smart concrete. Constr. Build. Mater..

[29] Dong W., Li W., Tao Z., Wang K. (2019). Piezoresistive properties of cement-based sensors: Review and perspective. Constr. Build. Mater..

[30] Xu D., Banerjee S., Wang Y., Huang S., Cheng X. (2015). Temperature and loading effects of embedded smart piezoelectric sensor for health monitoring of concrete structures. Constr. Build. Mater..

[31] Figarol A., Pourchez J., Boudard D., Forest V., Akono C., Tulliani J.-M., Lecompte J.-P., Cottier M., Bernache-Assollant D., Grosseau P. (2015). In vitro toxicity of carbon nanotubes, nano-graphite and carbon black, similar impacts of acid functionalization. Toxicol. Vitr..

[32] Hu N., Karube Y., Yan C., Masuda Z., Fukunaga H. (2008). Tunneling effect in a polymer/carbon nanotube nanocomposite strain sensor. Acta Mater..

[33] Barra G., Guadagno L., Vertuccio L., Simonet B., Santos B., Zarrelli M., Arena M., Viscardi M. (2019). Different Methods of Dispersing Carbon Nanotubes in Epoxy Resin and Initial Evaluation of the Obtained Nanocomposite as a Matrix of Carbon Fiber Reinforced Laminate in Terms of Vibroacoustic Performance and Flammability. Materials.

[34] Guadagno L., Raimondo M., Vertuccio L., Naddeo C., Barra G., Longo P., Lamberti P., Spinelli G., Nobile M. (2018). Morphological, rheological and electrical properties of composites filled with carbon nanotubes functionalized with 1-pyrenebutyric acid. Compos. Part B Eng..

[35] Kang I., Schulz M., Kim J.H., Shanov V., Shi D. (2006). A carbon nanotube strain sensor for structural health monitoring. Smart Mater. Struct..

[36] Wang J., Kazemi Y., Wang S., Hamidinejad M., Mahmud M.B., Pötschke P., Park C.B. (2020). Enhancing the electrical conductivity of PP/CNT nanocomposites through crystal-induced volume exclusion effect with a slow cooling rate. Compos. Part B Eng..

[37] Shen J., Champagne M.F., Yang Z., Yu Q., Gendron R., Guo S. (2012). The development of a conductive carbon nanotube (CNT) network in CNT/polypropylene composite films during biaxial stretching. Compos. Part A Appl. Sci. Manuf..

[38] Zetina-Hernández O., Duarte-Aranda S., May-Pat A., Canché-Escamilla G., Uribe-Calderón J.A., González-Chi P.I., Aviles F. (2013). Coupled electro-mechanical properties of multiwall carbon nanotube/polypropylene composites for strain sensing applications. J. Mater. Sci..

[39] Aviles F., Oliva-Avilés A.I., Cen-Puc M. (2018). Piezoresistivity, Strain, and Damage Self-Sensing of Polymer Composites Filled with Carbon Nanostructures. Adv. Eng. Mater..

[40] Zhao J., Dai K., Liu C., Zheng G., Wang B., Liu C., Chen J., Shen C. (2013). A comparison between strain sensing behaviors of carbon black/polypropylene and carbon nanotubes/polypropylene electrically conductive composites. Compos. Part A Appl. Sci. Manuf..

[41] Qu Y., Dai K., Zhao J., Zheng G., Liu C., Chen J., Shen C. (2013). The strain-sensing behaviors of carbon black/polypropylene and carbon nanotubes/polypropylene conductive composites prepared by the vacuum-assisted hot compression. Colloid Polym. Sci..

[42] Wang Z.-J., Kwon D.-J., Gu G.-Y., Kim H.-S., Kim D.-S., Lee C.-S., Devries K.L., Park J.-M. (2013). Mechanical and interfacial evaluation of CNT/polypropylene composites and monitoring of damage using electrical resistance measurements. Compos. Sci. Technol..

[43] Coppola B., Di Maio L., Scarfato P., Incarnato L. (2015). Use of polypropylene fibers coated with nano-silica particles into a cementitious mortar. Proceedings of the Polymer Processing with Resulting Morphology and Properties: Feet in the Present and Eyes at the Future, Proceedings of the GT70 International Conference.

[44] Eidan J., Rasoolan I., Rezaeian A., Poorveis D. (2019). Residual mechanical properties of polypropylene fiber-reinforced concrete after heating. Constr. Build. Mater..

[45] Kakooei S., Akil H.M., Jamshidi M., Rouhi J. (2012). The effects of polypropylene fibers on the properties of reinforced concrete structures. Constr. Build. Mater..

[46] Banthia N., Gupta R. (2006). Influence of polypropylene fiber geometry on plastic shrinkage cracking in concrete. Cem. Concr. Res..

[47] Patti A., Russo P., Acierno D., Acierno S. (2016). The effect of filler functionalization on dispersion and thermal conductivity of polypropylene/multi wall carbon nanotubes composites. Compos. B. Eng..

[48] Müller M.T., Krause B., Pötschke P. (2012). A successful approach to disperse MWCNTs in polyethylene by melt mixing using polyethylene glycol as additive. Polymer.

[49] Miquelard-Garnier G., Guinault A., Fromonteil D., Delalande S., Sollogoub C. (2013). Dispersion of carbon nanotubes in polypropylene via multilayer coextrusion: Influence on the mechanical properties. Polymer.

[50] Ezat G.S., Kelly A., Youseffi M., Coates P. (2019). Effect of screw configuration on the dispersion and properties of polypropylene/multiwalled carbon nanotube composite. Polym. Compos..

[51] Qureshi Y., Tarfaoui M., Lafdi K.K., Lafdi K. (2019). Real-time strain monitoring and damage detection of composites in different directions of the applied load using a microscale flexible Nylon/Ag strain sensor. Struct. Heal. Monit..

[52] Qureshi Y., Tarfaoui M., Lafdi K.K., Di Maio L. (2019). Real-time strain monitoring performance of flexible Nylon/Ag conductive fiber. Sens. Actuators A Phys..

[53] Aygun L., Kumar V., Weaver C., Gerber M., Wagner S., Verma N., Glisic B., Sturm J. (2020). Large-Area Resistive Strain Sensing Sheet for Structural Health Monitoring. Sensors.

[54] Ahuja P., Ujjain S.K., Urita K., Furuse A., Moriguchi I., Kaneko K. (2020). Chemically and mechanically robust SWCNT based strain sensor with monotonous piezoresistive response for infrastructure monitoring. Chem. Eng. J..

[55] Downey A., Pisello A.L., Fortunati E., Fabiani C., Luzi F., Torre L., Ubertini F., Laflamme S. (2019). Durability and weatherability of a styrene-ethylene-butylene-styrene (SEBS) block copolymer-based sensing skin for civil infrastructure applications. Sens. Actuators A Phys..

[56] Gerber M., Weaver C., Aygun L., Verma N., Sturm J., Glisic B. (2018). Strain Transfer for Optimal Performance of Sensing Sheet. Sensors.

[57] Ahuja P., Akiyama S., Ujjain S.K., Kukobat R., Vallejos-Burgos F., Futamura R., Hayashi T., Kimura M., Tomanek D., Kaneko K. (2019). A water-resilient carbon nanotube based strain sensor for monitoring structural integrity. J. Mater. Chem. A.

[58] Ye X., Jiawei M., Jie L., Ye X., Ma J., Li J. (2019). Development of a transparent and stretchable strain sensor based on the dry printing method. Mater. Res. Express.

[59] Min S.-H., Lee G.-Y., Ahn S.-H. (2019). Direct printing of highly sensitive, stretchable, and durable strain sensor based on silver nanoparticles/multi-walled carbon nanotubes composites. Compos. Part B Eng..

[60] Nayak L., Mohanty S., Nayak S.K., Ramadoss A. (2019). A review on inkjet printing of nanoparticle inks for flexible electronics. J. Mater. Chem. C.

[61] Anderson N., Szorc N., Gunasekaran V., Joshi S., Jursich G. (2019). Highly sensitive screen printed strain sensors on flexible substrates via ink composition optimization. Sens. Actuators A Phys..

[62] Xiang N., Zhang X., Li Y., Harkin-Jones E., Zheng Y., Wang L., Zhao C., Wang P. (2019). Enhanced performance of 3D printed highly elastic strain sensors of carbon nanotube/thermoplastic polyurethane nanocomposites via non-covalent interactions. Compos. Part B Eng..

[63] Ye W., Wu W., Hu X., Lin G., Guo J., Qu H., Zhao J. (2019). 3D printing of carbon nanotubes reinforced thermoplastic polyimide composites with controllable mechanical and electrical performance. Compos. Sci. Technol..

[64] Xiang D., Zhang X., Harkin-Jones E., Zhu W., Zhou Z., Shen Y., Li Y., Zhao C., Wang P. (2020). Synergistic effects of hybrid conductive nanofillers on the performance of 3D printed highly elastic strain sensors. Compos. Part A Appl. Sci. Manuf..

[65] Mora A., Verma P., Kumar S. (2020). Electrical conductivity of CNT/polymer composites: 3D printing, measurements and modeling. Compos. Part B Eng..

[66] Acierno S., Barretta R., Luciano R., De Sciarra F.M., Russo P. (2017). Experimental evaluations and modeling of the tensile behavior of polypropylene/single-walled carbon nanotubes fibers. Compos. Struct..

[67] Naeem F., Lee H.K., Kim H.K., Nam I.W. (2017). Flexural stress and crack sensing capabilities of MWNT/cement composites. Compos. Struct..

[68] Ozmaian M., Naghdabadi R. (2013). Semi-conducting carbon nanotube as variable capacitor. Phys. E.

[69] Yetgin S.H. (2019). Effect of multi walled carbon nanotube on mechanical, thermal and rheological properties of polypropylene. J. Mater. Res. Technol..

[70] Assouline E., Lustiger A., Barber A., Cooper C.A., Klein E., Wachtel E., Wagner H.D. (2003). Nucleation ability of multiwall carbon nanotubes in polypropylene composites. J. Polym. Sci. Part B Polym. Phys..

[71] Leelapornpisit W., Cole K.C., Denault J., Simard B., Ton-That M.-T., Perrin-Sarazin F. (2005). Effect of carbon nanotubes on the crystallization and properties of polypropylene. J. Polym. Sci. Part B Polym. Phys..

[72] Das D., Satapathy B.K. (2014). Designing tough and fracture resistant polypropylene/multi wall carbon nanotubes nanocomposites by controlling stereo-complexity and dispersion morphology. Mater. Des..

[73] Coppola B., Cappetti N., Di Maio L., Scarfato P., Incarnato L. (2018). 3D Printing of PLA/clay Nanocomposites: Influence of Printing Temperature on Printed Samples Properties. Materials.

[74] Kashiwagi T., Grulke E., Hilding J., Harris R., Awad W., Douglas J. (2002). Thermal degradation and flammability properties of poly (propylene)/carbon nanotube composites. Macromol. Rapid Commun..

[75] Coppola B., Scarfato P., Incarnato L., Di Maio L. (2017). Morphology Development and Mechanical Properties Variation during Cold-Drawing of Polyethylene-Clay Nanocomposite Fibers. Polymers.

[76] Stan F., Sandu L.I., Fetecau C., Rosculet R. (2017). Effect of Reprocessing on the Rheological, Electrical, and Mechanical Properties of Polypropylene/Carbon Nanotube Composites. J. Micro Nano-Manuf..

[77] Lecocq H., Garois N., Lhost O., Girard P.F., Cassagnau P., Serghei A. (2020). Polypropylene/carbon nanotubes composite materials with enhanced electromagnetic interference shielding performance: Properties and modeling. Compos. B. Eng..

[78] Hu N., Masuda Z., Yamamoto G., Fukunaga H., Hashida T., Qiu J. (2008). Effect of fabrication process on electrical properties of polymer/multi-wall carbon nanotube nanocomposites. Compos. Part A Appl. Sci. Manuf..

[79] Cipriano B.H., Kota A.K., Gershon A.L., Laskowski C.J., Kashiwagi T., Bruck H., Raghavan S.R. (2008). Conductivity enhancement of carbon nanotube and nanofiber-based polymer nanocomposites by melt annealing. Polymer.

[80] Yoo D.-Y., You I., Lee S.-J. (2018). Electrical and piezoresistive sensing capacities of cement paste with multi-walled carbon nanotubes. Arch. Civ. Mech. Eng..

[81] Camacho-Ballesta C., Zornoza E., Garces P. (2016). Performance of cement-based sensors with CNT for strain sensing. Adv. Cem. Res..

[82] Sasmal S., Ravivarman N., Sindu S.B., Vignesh K. (2017). Electrical conductivity and piezo-resistive characteristics of CNT and CNF incorporated cementitious nanocomposites under static and dynamic loading. Compos. Part A Appl. Sci. Manuf..

[83] Buasiri T., Cwirzen H.-, Krzeminski L., Cwirzen A., Habermehl-Cwirzen K. (2019). Piezoresistive Load Sensing and Percolation Phenomena in Portland Cement Composite Modified with In-Situ Synthesized Carbon Nanofibers. Nanomaterials.

[84] Li H., Xiao H.-G., Ou J.-P. (2006). Effect of compressive strain on electrical resistivity of carbon black-filled cement-based composites. Cem. Concr. Compos..

[85] Noiseux-Lauze G., Akhras G. Structural health monitoring using smart nano cement sensors. Proceedings of the International workshop on smart materials, structures NDT for the energy industry.

[86] Galao O., Baeza F., Zornoza E., Garces P. (2017). Carbon Nanofiber Cement Sensors to Detect Strain and Damage of Concrete Specimens Under Compression. Nanomaterials.

[87] Yoo D.-Y., You I., Zi G., Lee S.-J. (2019). Effects of carbon nanomaterial type and amount on self-sensing capacity of cement paste. Measurement.

[88] Yoo D.-Y., You I., Lee S.-J. (2017). Electrical Properties of Cement-Based Composites with Carbon Nanotubes, Graphene, and Graphite Nanofibers. Sensors.

[89] Loh K.J., González J. (2015). Cementitious Composites Engineered with Embedded Carbon Nanotube Thin Films for Enhanced Sensing Performance. J. Phys. Conf. Ser..

[90] Zhan M., Pan G., Zhou F., Mi R., Shah S.P. (2020). In situ-grown carbon nanotubes enhanced cement-based materials with multifunctionality. Cem. Concr. Compos..

[91] Yoo D.Y., You I., Youn H., Lee S.J. (2018). Electrical and piezoresistive properties of cement composites with carbon nanomaterials. J. Compos. Mater..

[92] Azhari F., Banthia N. (2012). Cement-based sensors with carbon fibers and carbon nanotubes for piezoresistive sensing. Cem. Concr. Compos..

[93] Rao R., Sindu B., Sasmal S. (2020). Synthesis, design and piezo-resistive characteristics of cementitious smart nanocomposites with different types of functionalized MWCNTs under long cyclic loading. Cem. Concr. Compos..

[94] Han B., Zhang L., Sun S., Yu X., Dong X., Wu T., Ou J. (2015). Electrostatic self-assembled carbon nanotube/nano carbon black composite fillers reinforced cement-based materials with multifunctionality. Compos. Part A Appl. Sci. Manuf..

